# Supplementary material for: Immune Activation and Inflammatory Response Mediated by the NOD/Toll-like Receptor Signaling Pathway—The Potential Mechanism of Bullfrog (Lithobates catesbeiana) Meningitis Caused by Elizabethkingia miricola
Source: Int J Mol Sci. 2023 Sep 26;24(19):14554. doi: 10.3390/ijms241914554 (PMC10572524; doi:10.3390/ijms241914554)
Supplement: Supplementary file 1 [file ijms-24-14554-s001.zip › Supplementary materials (Figure).pdf]

Supplementary materials (Figures)

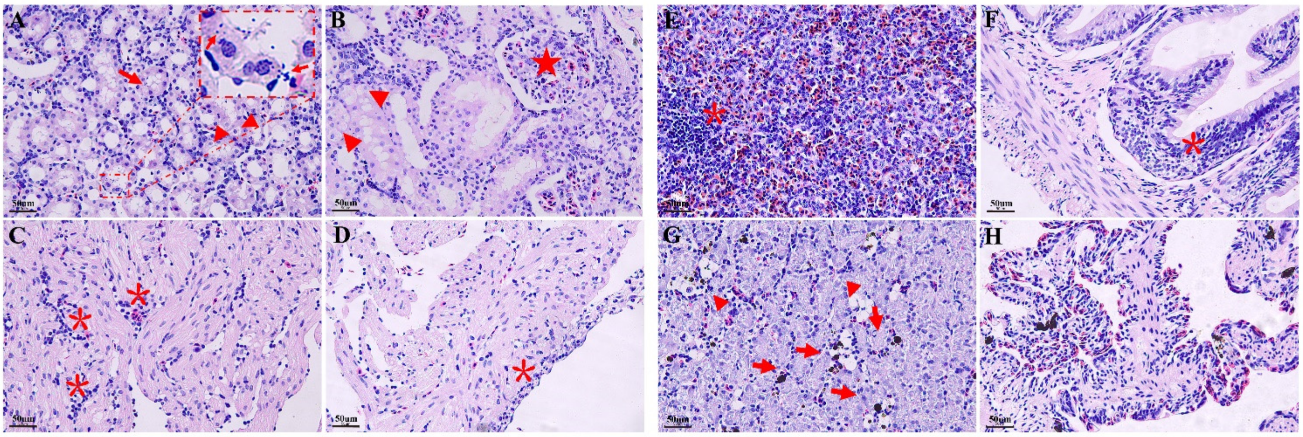

Figure S1. Histopathological changes in bullfrogs infected with *Elizabethkingia miricola*. **(A-B)** Enlarged and vacuolated renal tubular epithelial cells (no trailing arrow), some cell necrosis (trailing arrow), and dilated renal capsule lumen (pentagram). **(C-F)** Inflammatory cell infiltration in the heart, intestines and spleen (asterisks). **(G)** The liver shows more brown pigmentation (with trailing arrow) and vacuolar degeneration of hepatocytes (without trailing arrow). **(H)** Slight bruising in the alveolar microvasculature.

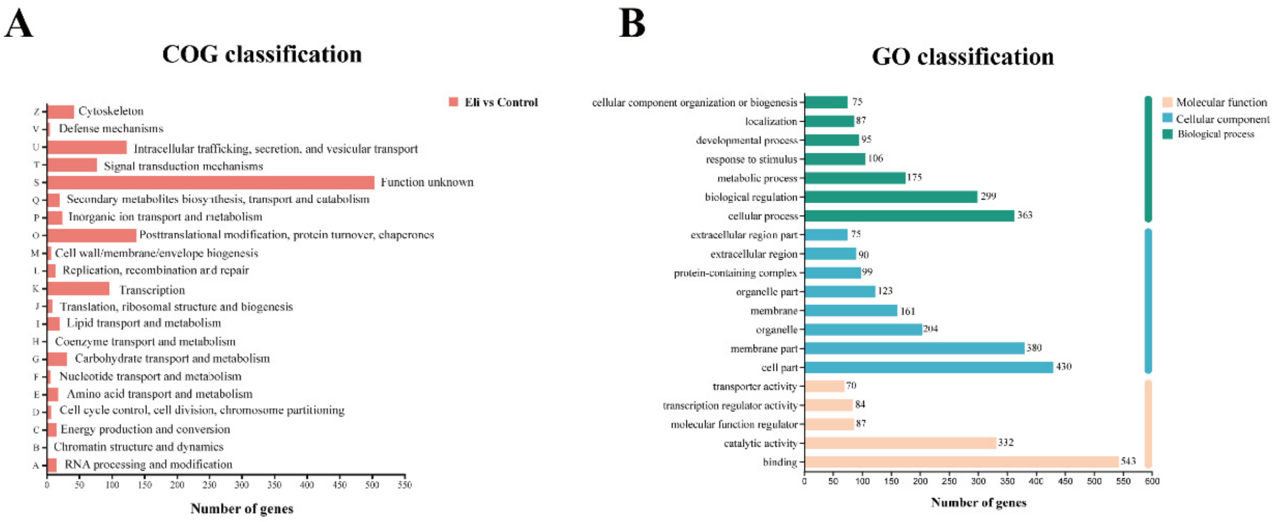

Figure S2. Functional classification of differentially expressed genes (DEGs) between the control and Eli groups. **(A)** COG, **(B)** GO annotation of DEGs.

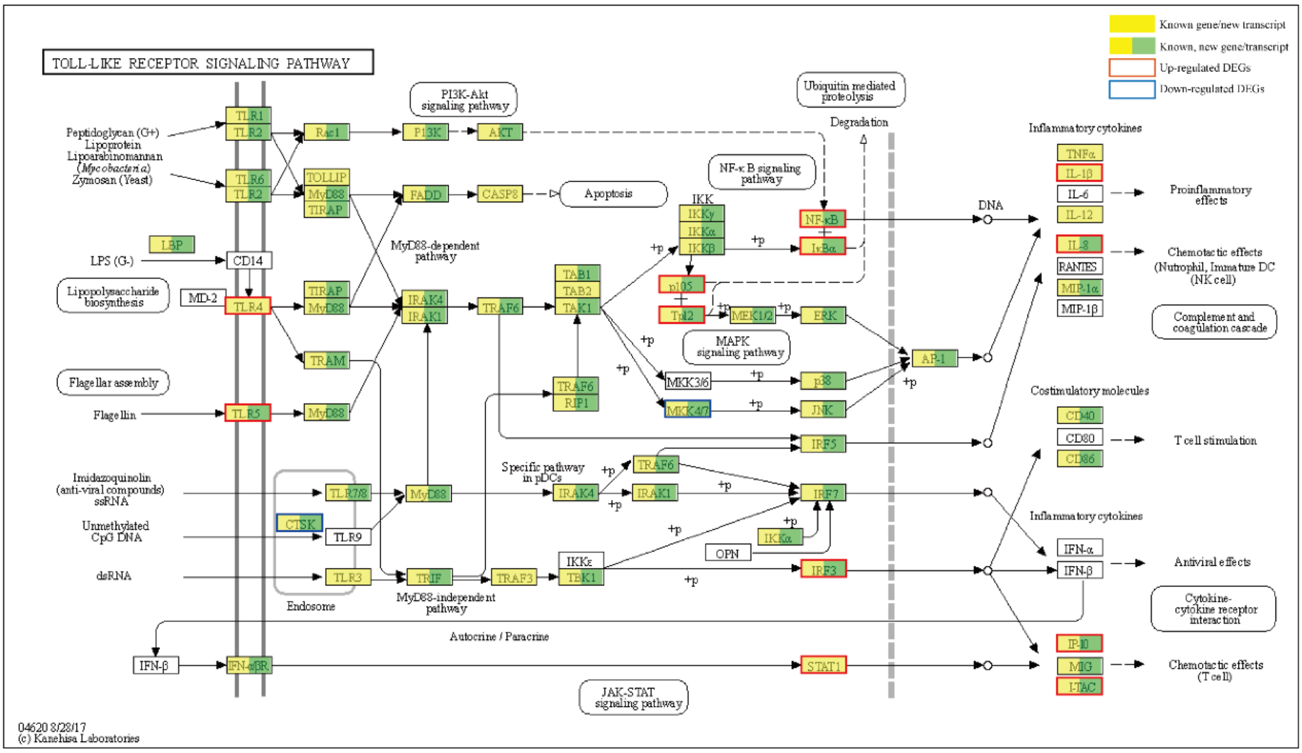

Figure S3. DEGs in Toll-like receptor signaling pathway.
