# Supplementary material for: Immune Activation and Inflammatory Response Mediated by the NOD/Toll-like Receptor Signaling Pathway—The Potential Mechanism of Bullfrog (Lithobates catesbeiana) Meningitis Caused by Elizabethkingia miricola
Source: Int J Mol Sci. 2023 Sep 26;24(19):14554. doi: 10.3390/ijms241914554 (PMC10572524; doi:10.3390/ijms241914554)
Supplement: Supplementary file 1 [file ijms-24-14554-s001.zip › Supplementary materials (Table).pdf]

Supplementary materials (Tables)

Table S1. The results about the comparison of clean reads with reference genome.

Sample	Total reads	Total mapped	Multiple mapped	Uniquely mapped
**Control-1**	54,645,342	43,656,324(79.89%)	1,424,065(2.61%)	42,232,259(77.28%)
**Control-2**	54,541,358	43,677,586(80.08%)	1,414,112(2.59%)	42,263,474(77.49%)
**Control-3**	62,634,710	50,558,613(80.72%)	1,720,655(2.75%)	48,837,958(77.97%)
**Eli-1**	65,771,358	51,336,621(78.05%)	1,673,533(2.54%)	49,663,088(75.51%)
**Eli-2**	59,997,100	47,546,302(79.25%)	1,478,613(2.46%)	46,067,689(76.78%)
**Eli-3**	61,569,246	48,970,883(79.54%)	1,518,183(2.47%)	47,452,700(77.07%)

Table S2. Histopathological assessment. An importance factor (ωif), ranging from 1 to 3, was assigned to every change (1—minimal pathological importance: the lesion is easily reversible as exposure to irritants ends; 2—moderate pathological importance: the lesion is reversible in most cases if the stressor is neutralized; and 3—marked pathological importance: the lesion is generally irreversible, leading to partial or total loss of the organ function [33]).

Organ (Org)	Functional unit of the tissue (t)	Alteration (alt)	Importance factor (ωif)
**Brain**	Meninges/brain parenchyma	structural alterations	1
**Eye**	Retina/cornea	hemorrhage	1
**Kidney**	Tubule/glomerulus	deposits	1
**Heart**	myocardium	edema	1
**Liver**	Liver tissue/interstitial tissue	hyperplasia	2
**Intestinal**	Mucosa layer	infiltration	2
**Spleen**	Interstitial tissue	Atrophy	2
**Lung**	Alveolar/capillaries	necrosis	3

Table S3. Primer sequences in this study.

Gene name	Primer sequence (5′-3′)	Size (bp)	GenBank accession number
NFKB	F: AGGAACAAGATCATCATAGAGGAC<br>R: TCAGAAAGAGGGGCCAAGTG	157	XM_040329914.1
COX2	F: CGCAGCCTCCCCCATTATAG<br>R: GATGCGAAGAGATGGGAGGG	217	ON746668.1
IL-8	F: ACCCTTACCCTCTTCCTGCT<br>R: TCAGCTTCACACACTGGCA	95	XM_040335899.1
TLR4	F: ACACCTTCAAGGCTCGCTAC<br>R: ATGATGGGTCGCCATCTTCC	278	XM_040322748.1
CARD9	F: AAAGGAGGCCACCATTAGGC<br>R: TCGCATGACAGCATTGGTCT	190	XM_040324146.1
β-actin	F: TGCTCTGGACTTTGAGCAGG<br>R: ACTTGTCCATCAGGCAGCTC	84	XM_040357114.1

F, forward primer; R, reverse primer. *NFKB*: nuclear factor kappa B, *COX2*: Cyclooxygenase 2, *IL-8*: interleukin-8-like, *TLR4*: toll like receptor 4, *CARD9*: caspase recruitment domain family member
